# Insights into fungal communities in composts revealed by 454-pyrosequencing: implications for human health and safety

**DOI:** 10.3389/fmicb.2013.00164

**Published:** 2013-06-13

**Authors:** Vidya De Gannes, Gaius Eudoxie, William J. Hickey

**Affiliations:** ^1^Department of Food Production, Faculty of Food and Agriculture, University of the West Indies, St. Augustine CampusSt. Augustine, Republic of Trinidad and Tobago; ^2^O.N. Allen Laboratory for Soil Microbiology, Department of Soil Science, University of Wisconsin-MadisonMadison, WI, USA

**Keywords:** compost, fungi, pathogens, 454-pyrosequencing, diversity

## Abstract

Fungal community composition in composts of lignocellulosic wastes was assessed *via* 454-pyrosequencing of ITS1 libraries derived from the three major composting phases. *Ascomycota* represented most (93%) of the 27,987 fungal sequences. A total of 102 genera, 120 species, and 222 operational taxonomic units (OTUs; >97% similarity) were identified. Thirty genera predominated (*ca.* 94% of the sequences), and at the species level, sequences matching *Chaetomium funicola* and *Fusarium oxysporum* were the most abundant (26 and 12%, respectively). In all composts, fungal diversity in the mature phase exceeded that of the mesophilic phase, but there was no consistent pattern in diversity changes occurring in the thermophilic phase. Fifteen species of human pathogens were identified, eight of which have not been previously identified in composts. This study demonstrated that deep sequencing can elucidate fungal community diversity in composts, and that this information can have important implications for compost use and human health.

## Introduction

Compost is a dynamic and complex habitat wherein microorganisms play fundamental roles. Bacterial communities in these systems have been extensively studied (Tiquia, 2005; Takaku et al., 2006; Thummes et al., 2007; Partanen et al., 2010; De Gannes et al., 2012, 2013). However, fungi are another major group of compost microbes that contribute essential decomposition processes, particularly the initial degradation of plant polymers (Sharma, 1989; Floudas et al., 2012). Relatively little is known, however, about the composition, diversity, and succession patterns of fungal communities in compost.

While compost is valued as a soil amendment, potential health hazards have also been recognized, as some saprotrophic fungi that colonize these materials are also opportunistic human pathogens. The primary risk is exposure to bio-aerosols containing compost-derived fungi, which in humans can cause mycoses of the lungs, skin, ears, sinuses, or bone marrow (Bunger et al., 2000). Most attention has focussed on *Aspergillus fumigatus*, the most commonly identified fungal pathogen in composts (Ryckeboer et al., 2003; Anastasi et al., 2005; Dehghani et al., 2012). But, other pathogens have also been reported, including *Candida tropicalis, Candida krusei* (Bonito et al., 2010), *Scytalidium lignicola*, and *Alternaria alternata* (Anastasi et al., 2005). The spectrum of saprotrophic fungi that may also be opportunistic human pathogens is much greater than the foregoing list, but their occurrence in composts has not been extensively explored.

Molecular analyses have the potential to provide new insights into the composition of fungal communities in compost. However, to date, such analyses have indicated that fungal diversity in these systems is low. For example, Tiquia (2005) applied 18S rDNA terminal-restriction fragment length analysis for profiling of manure composts, and identified a total of 18 unique fragments, presumably indicative of 18 different phylotypes. Also, 10 genera were identified from libraries of 18S rRNA genes that were cloned from a model compost of dog food-wood chips (Hansgate et al., 2005). Hultman et al. (2010) created libraries of internal transcribed spacer (ITS) region sequences from municiple waste compost, and identified 166 OTUs (≥99% similarity) from *ca*. 2900 clones. It's unclear if the relatively low diversity indicated by these studies accurately reflects the communities, or if it results from a limited depth of analysis. Similarly, the dynamics of fungal communities as a function of compost phase are as yet ill-defined. In principle, since most fungi are mesophiles, a reduction in their diversity might be expected as compost heat to thermophilic temperatures. But, to date, results from molecular analyses do not consistently support that concept (Tiquia, 2005; Bonito et al., 2010) and it's unclear as to the extent to which the divergent finding reflects variation in compost properties (e.g., level and duration of heating) *vs*. limitations of the molecular analyses applied.

Recently, knowledge of fungal communities in soils has been expanded with the advent of the use of high-throughput sequencing technologies such as 454-pyrosequencing. Compared to traditional approaches based on cloning and Sanger sequencing in terms of cost, time, and number of sequences obtained, 454-pyrosequencing has proven to be a powerful alternative for the identification of a greater depth of taxa (Tedersoo et al., 2010). For example, Lentendu et al. (2011) demonstrated an unprecedented depth of fungal diversity in forest soil, and illustrated the strong spatial heterogeneity of fungal communities in these soils. Buee et al. (2009) were able to identify rare individuals (OTUs), which could be correlated with ecological parameters. Pyrosequencing of the nuclear ribosomal ITS region revealed fungal communities of a cork oak forest soil were distinct from those described in other soils (Orgiazzi et al., 2012). Pyrosequencing also proved useful in the examination of fungal communities related to plant disease in agriculture soils (Xu et al., 2012).

While high through-put sequencing has greatly advanced our understanding of fungal communities in soil, its application to assess these organisms in compost has not been reported. Pyrosequencing analysis could provide new insights into the composition of fungal communities in compost, and how their evolution compares to the chemical evolution of the composts. Furthermore, deep sequencing could expand the diversity of human pathogens present in compost. Thus, the goal of our study was to obtain in-depth sequence information to assess diversity and dynamics of fungal communities in three composts of lignocellulosic agricultural wastes. Fungal composition was assessed *via* high throughput 454-pyrosequencing of ITS1 libraries derived from each of the three major phases of the compost process. We hypothesized that community structure of each system is unique throughout the composting process, and that these systems do not converge on a common composition in the finished product. We also hypothesized that deep sequencing of these communities would reveal a greater diversity of potential pathogens than has been indicated in the literature to date.

## Materials and methods

### Compost composition and analyses

The compost study was described previously in De Gannes et al. (2012). In-vessel composting was done in rotary drums, in a randomized 3 × 2 factorial design with six treatments and two replications per treatment. Substrates used for composting were agricultural wastes (*viz*. rice straw, sugar cane bagasse, coffee hulls) mixed with either cow- or sheep-manure to obtain a C:N of 25–35:1.

### Pyrosequencing and data analyses

Sampling and DNA extraction was described previously in De Gannes et al. (2012). Briefly, the composts were sampled at the mesophilic stage (Day 0), thermophilic stage (Day 2 for rice straw composts, Day 3 for coffee hulls composts, no sample for sugar cane bagasse) and completion of the study (mature stage, Day 82). Aliquots of the treatment replicates were pooled, and an aliquot from each of these samples was then used for PCR. For the mature phase of the bagasse and coffee composts, two replicate aliquots were used in PCR, giving a total of 10 samples analyzed by pyrosequencing. While fungal DNA recovered could have originated from spores or vegetative cells, the latter are more to be the primary source as the extraction method employed (Power Soil DNA extraction kit; MoBio), lacks the more vigorous physical disruption techniques that are often needed for rupture of fungal spores.

Fungal DNA was amplified by using primers ITS1F and ITS2 (Table S1) to generate *ca*. 400 bp fragments of the nuclear ribosomal ITS1 region. Each forward primer contained the A linker for sequencing, and one of 10 unique Roche multiplex identifiers (Table S1). Thermal cycling parameters were: 94°C for 4 min, 30 s at 94°C (30 cycles), 50°C min, 72°C for 90 s, and a final extension of 10 min at 72°C. Replicate PCR was done with the mature phase of the bagasse and coffee as described previously (De Gannes et al., 2012). However, technical issues occurring during sequencing resulted in no useable data being returned from one of the replicate bagasse samples. Amplicon concentrations were measured with a Qubit fluorometer (Invitrogen, Grand Island, NY), the libraries were pooled in equimolar concentrations and then cleaned by 5× passage over AMPure beads (Beckman Coulter, Brea, CA). Clonal amplified *via* emulsion PCR was done with the GS FLX Titanium Lib-L LV emPCR Kit (Roche Applied Science, Indiananpolis, IN) following the manufacturer's recommendations, with the exception that the amplification primer was used at 25% of the recommended volume. A DNA library was created by using a ratio of 1 DNA molecule per capture bead. Clonally amplified DNA was collected and enriched according to the manufacturer's protocol, and the number of enriched DNA beads determined with a CASY Model DT cell counter (Roche Applied Science). Beads were added to wells of a GS FLX Titanium PicoTiterPlate fitted with a 2-region gasket, and sequenced using a GS FLX Titanium Sequencing Kit XL+ according to the manufacturer's instructions. Image analysis and signal processing were done through the shotgun/paired end signal processing pipeline in GS Run Processor v. 2.6 (Roche Applied Science). The raw data set prior to denoising was 36,314 sequences.

Data processing was done by using the ITS Pipeline (Nilsson et al., 2009). Sequence reads of <100 bp were deleted from the data set, and the ITS extractor used to obtain ITS1 data free of sequences mapping to the 5.8S, 18S, or 28S rRNA genes. The TIGR Gene Clustering Indices tool was used to create OTUs at 97% similarity with reference to the UNITE (Abarenkov et al., 2010) and INSD (Karsch-Mizrachi et al., 2011) databases. Species level identities were confirmed by manual BLAST against the Genbank non-redundant data base followed by inspection to verify a full length match of ≥97% to a sequence of a *bona fide* fungal culture. Denoising of the dataset, rarefaction analysis by a radomization procedure that included singletons was done with QIIME v. 1.5.0 (Caporaso et al., 2010). Rank abundance was calculated in Excel, and plotted with Prism v. 5.0 (GraphPad Software, Inc., La Jolla, CA). The relative abundance of a given taxon was equated with relative abundance of sequence reads, assuming the caveats delineated by Amend et al. (2010). Furthermore, while the ITS region is widely used for species delimitation, it is also recognized that ITS sequences are insufficient to resolve some fungal species and species complexes, and species assignments are made recognizing that limitation. Also, either ITS1 or ITS2 could be used for analysis of fungal communities, each has benefits and drawbacks and the selection largely dependent upon the goals of the study (Bazzicalupo et al., 2013). For the present work, ITS1 was selected, as there is a somewhat larger use of that region in the literature, and to which the present work would be comparable (Chen et al., 2001; Narutaki et al., 2002; Hinrikson et al., 2005; Nilsson et al., 2008).

### Sequence accession numbers

Sequence data have been deposited in National Center for Biotechnology Information, Sequence Read Archive (SRX079878).

## Results

### Chemical properties of composts

Chemical properties of the composts were discussed previously in De Gannes et al. (2012). Briefly, by day 2, the rice composts attained a thermophilic temperature of *ca*. 57°C, and remained at that temperature for 3–4 days before the onset of cooling. The coffee composts attained a peak thermophilic temperature of 64°C by day eight, but maintained thermophilic temperature for *ca*. 5 days. The sugar cane bagasse compost reached a peak temperature of *ca*. 38°C, which was not considered a thermophilic stage (minimum 50°C). All composts showed reductions in total organic carbon (TOC) with time. However, rice showed the fastest rate of decrease. At the end of the study (day 82), TOC for rice, bagasse, and coffee were *ca*. 22 mg/kg, ca. 26 mg/kg, and *ca*. 28 mg/kg, respectively. The rice compost had a final C:N of 11, while that of the coffee and bagasse was *ca*. 15 and 17, respectively. The dynamics of nitrogen transformations also varied in these composts. For the bagasse and rice, nitrate increased steadily during the course of the study and by its end, the nitrate content for bagasse was *ca*. 100 mg/kg and that for rice was *ca*. 120 mg/kg. Nitrate accumulation for the coffee, however, was slower reaching 30 mg/kg at the end of the study. Each compost also showed a unique pH profile. The pH of coffee was initially at 5–6 but increased to *ca*. 7.4 by the end of the study. Bagasse also had an initial pH of 5–6, which spiked to pH 7–8 at the third week and then returned to pH 5–6 at the end (day 82). Contrastingly, the rice was initially alkaline (pH 8), but by the end of the study decreased to a pH of 6–7.

### Library composition and fungal diversity

**Figure 1:**
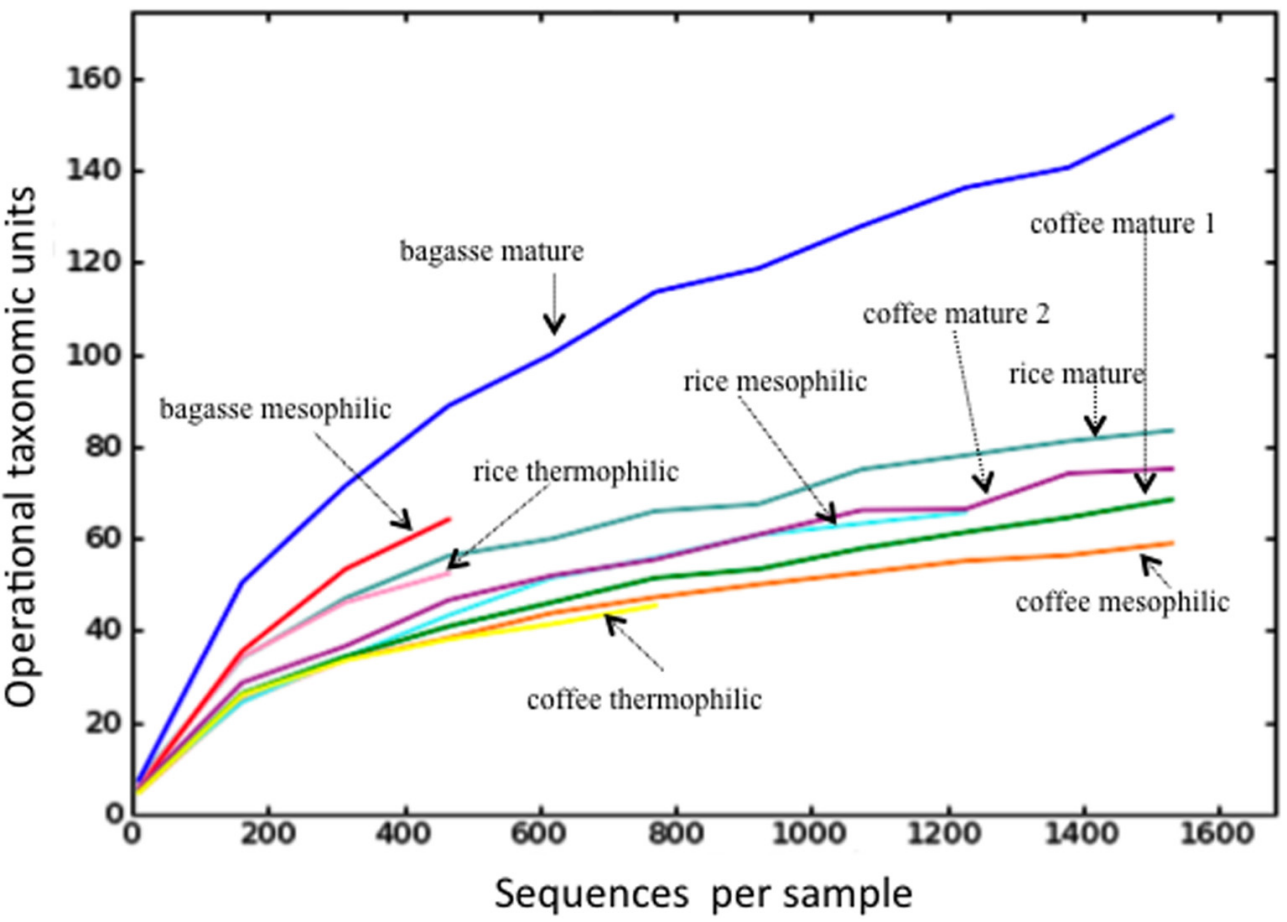
**Rarefaction analyses of the compost libraries**. Each curve illustrates the cumulative number of operational taxonomic units at a phylogenetic distance of 0.03 for the indicated compost. For coffee, replicate libraries from the mature phase are indicated by “1” and “2.”

A total of 28,145 sequences were returned *via* ITS Pipeline processing and, after removal of 158 sequences matching animal taxa, the fungal sequences totaled 27,987 (Table 1, Table S2). While it's possible that the primers could also amplify the ITS from plants, no plant sequences were identified. A total of 222 OTUs were identified with the number in any given library ranging from 39 to 149 (Table 1). Only six OTUs were identified in all libraries (Table S2), which matched to *Chaetomium funicola* (OTU 350), *Aspergillus oryzae* (OTU 38), *Thermomyces lanuginosus* (OTU 123, OTU 59), and *Fusarium oxysporum* (OTU 506, OTU 83). Rarefaction curves for most libraries approached a plateau or were asymptotic (Figure 1). Those for mesophilic bagasse and mature phase rice were truncated because of the limited number of sequences obtained, nevertheless, these samples paralleled curves of the other more extensively sequenced libraries, suggesting a similar trend. Overall, rarefaction analyses indicated that the libraries provided an adequate sampling of fungal diversity in the composts.

**Table 1:** **Characteristics of amplicon libraries^a^**.

	**BM**	**BMT**	**CM**	**CT**	**CMT.1**	**CMT.2**	**RM**	**RT**	**RMT**	**Total**
No. of sequences	522	6557	1509	7225	5983	892	1340	580	3537	28156
No. of OTU	44	148	39	88	88	92	50	49	78	229^b^

a Abbreviations: B, Bagasse; C, Coffee; R, Rice; M, Mesophilic; T, Thermophilic; MT, Mature.

b Total number of OTU determined for the entire database (see Table S2).

**Figure 2:**
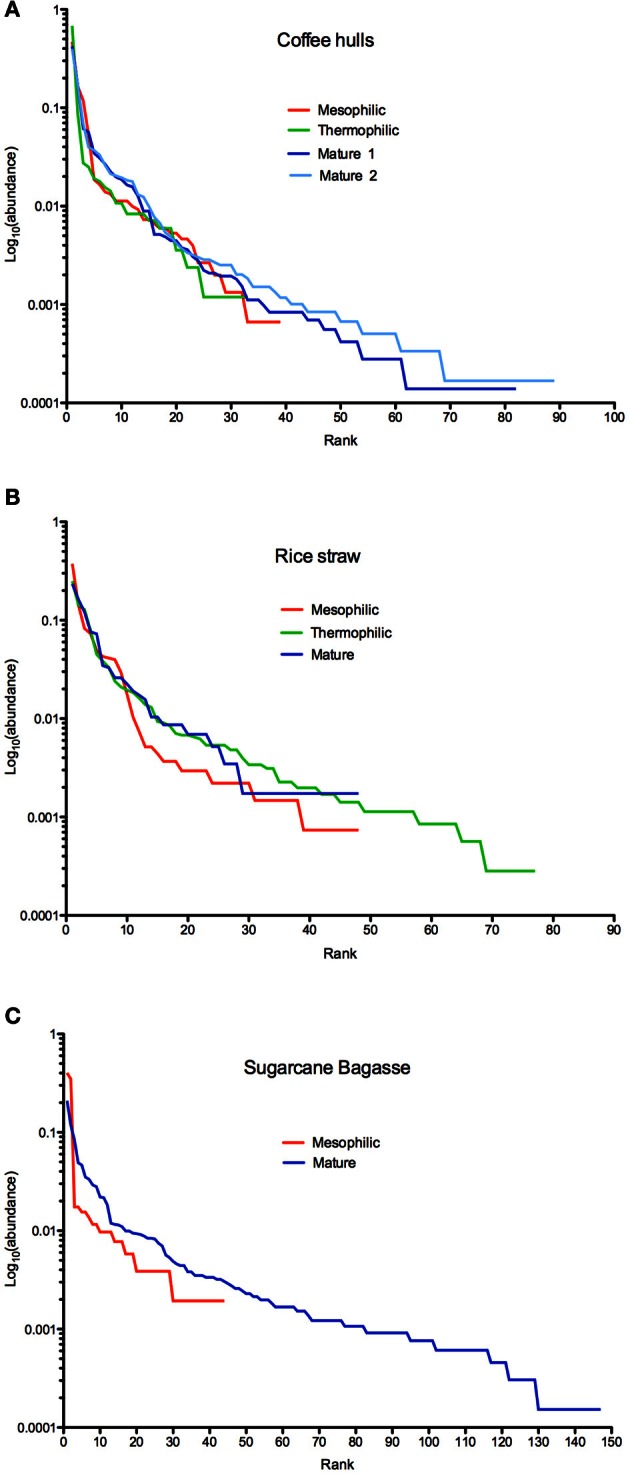
**Rank abundance plots of fungal community composition in the coffee (Panel A), rice (Panel B), and bagasse (Panel C) composts**.

Rank-abundance plots displayed contrasting patterns of species richness and at the same time, highlighted evenness among assemblages (Tokeshi, 1993; Smith and Wilson, 1996) (Figures 2A–C). In the coffee compost, the community of the thermophilic phase showed the lowest species evenness (lowest curve) and also the lowest species richness (shortest curve). By the mature phase, both the mesophilic and thermophilic parameters increased (Figure 2A). All phases were dominated by one or two OTU, as illustrated in a sharp drop in abundance from the top-ranks (Figure 2A). But, the gap between the dominant OTU and the next tier of OTUs narrowed in the mature phase, indicating proliferation of sub-dominant groups. In contrast, for the rice compost, species evenness increased after the mesophilic phase to similar levels in the thermophilic and mature stages (Figure 2B). Species richness, however, reached a maximum in the thermophilic phase, and in the mature phase decreased to levels similar to those occurring at the beginning of the study. The bagasse system showed the greatest alterations in community composition, and by the end of the study attained the highest level of species richness observed in any of the composts.

### Taxonomic composition of communities

**Figure 3:**
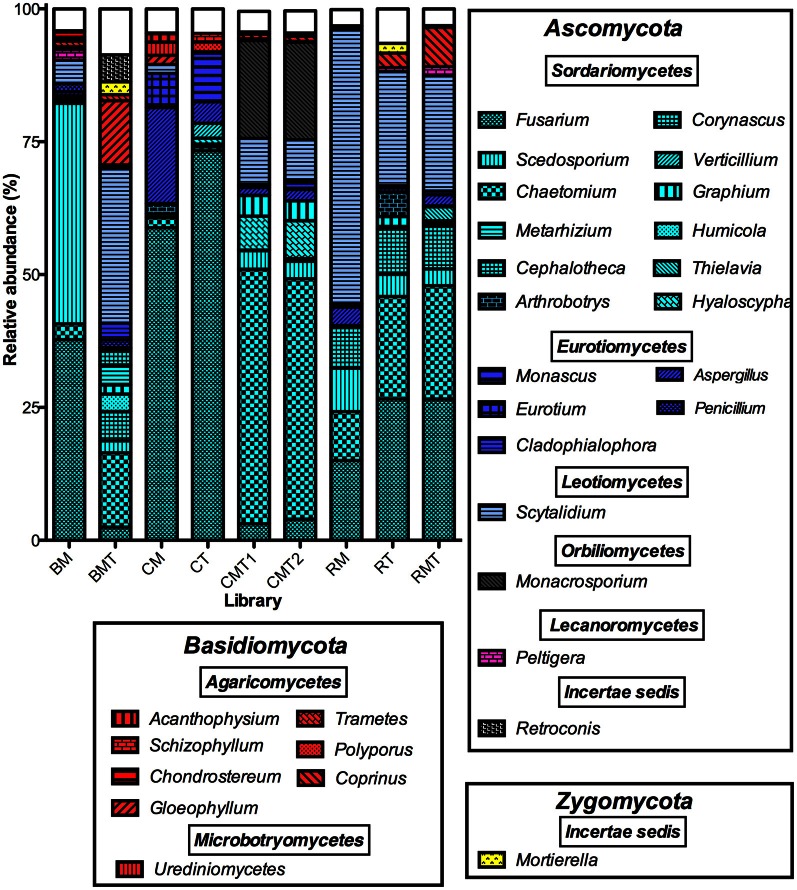
**Composition of compost sequence libraries illustrating the distribution of 30 genera that accounted for 94% of the sequences in the entire dataset**. The segments composing each bar are the number of sequences matching the indicated genus normalized to the total number of sequences in each library. Genera are sorted by phylum (boxes in figure), bars are color-coded by class as indicated. White boxes are the sum of all other genera in each library. Abbreviations are as follows: BM, bagasse mesophilic phase; BMT, bagasse mature phase; CM, coffee mesophilic phase; CT, coffee thermophilic phase; CMT1, coffee mature phase replicate 1; CMT2, coffee mature phase replicate 2; RM, rice mesophilic phase; RT, rice thermophilic phase; RMT, rice mature phase.

A total of 102 genera and 120 species were identified (Table S2) with 30 genera accounting for *ca.* 94% of the sequences (Table 2, Figure 3). In some instances, more than one OTU corresponded to the same species (Table S2). Most of these species were from phylum *Ascomycota* and class *Sordariomycetes*, which was the predominant phylum and class in all compost types and phases (93% of the sequences, Table S2). The *Basidiomycota* and *Zygomycota* accounted for 6 and 1% of the total sequences, respectively. At the species level, sequences that matched to *Chaetomium funicola* and *Fusarium oxysporum* were the most abundant, accounting for 26 and 12%, respectively of all sequence reads (Table S2). It should be noted that only a few species of fungi are known thermophiles and can survive the thermophilic temperature stage. In this regard only few fungi identified in this study are known thermophiles and they include *Aspergillus fumigatus, Scytalidium thermophilum*, and *Thermomyces lanuginosus*. Thus, this reason (i.e., small number of thermophilic fungi detected) and also the dynamic shifts in fungal diversity from the beginning to the end of the composting study for each system (even though it is difficult to discern whether or not the fungal communities detected were living organisms or resting spores), suggests that there was turn over in the DNA. In this regard, if they were merely resting spores, then those fungi which were detected alone in the beginning of the study would have been detected alone throughout the experiment.

**Table 2:** **Most abundant fungal genera identified for the predominant genera composing compost libraries^a^**.

**Phylum**	**Class**	**Order**	**Family**	**Genus**	**% Total^b^**
*Ascomycota*	*Sordariomycetes*	*Sordariales*	*Chaetomiaceae*	*Chaetomium*	28.5
*Ascomycota*	*Leotiomycetes*	*Helotiales*	*Incertae sedis*	*Scytalidium*	16.3
*Ascomycota*	*Sordariomycetes*	*Hypocreales*	*Nectriaceae*	*Fusarium*	12.1
*Ascomycota*	*Orbiliomycetes*	*Orbiliales*	*Orbiliaceae*	*Monacrosporium*	8.7
*Ascomycota*	*Sordariomycetes*	*Microascales*	*Microascaceae*	*Scedosporium*	3.8
*Ascomycota*	*Leotiomycetes*	*Helotiales*	*Hyaloscyphaceae*	*Hyaloscypha*	3.3
*Basidiomycota*	*Agaricomycetes*	*Gleophyllales*	*Cloeophyllaceae*	*Gleophyllum*	3
*Ascomycota*	*Sordariomycetes*	*Sordariales*	*Chaetomiaceae*	*Corynascus*	3
*Ascomycota*	*Sordariomycetes*	*Microascales*	*Microascaceae*	*Graphium*	2.4
*Ascomycota*	*Eurotiomycetes*	*Eurotiomycetes*	*Eurotiales*	*Aspergillus*	2.2
*Ascomycota*	*Eurotiomycetes*	*Eurotiales*	*Monascaceae*	*Monascus*	1.8
*Ascomycota*	*Incerate sedis*	*Incerate sedis*	*Incerate sedis*	*Retroconis*	1.2
*Zygomycota*	*Incerate sedis*	*Mottierellales*	*Mortierellaceae*	*Mortierella*	1
*Basidiomycota*	*Agaricomycetes*	*Agaricales*	*Agaricaceae*	*Coprinus*	0.9
*Ascomycota*	*Sordariomycetes*	*Hypocreales*	*Clavicipitaceae*	*Metarhizium*	0.8
*Ascomycota*	*Sordariomycetes*	*Sordariales*	*Chaetomiaceae*	*Humicola*	0.8
*Ascomycota*	*Sordariomycetes*	*Chaetothyriales*	*Herpotrichiellaceae*	*Cladophialophora*	0.7
*Ascomycota*	*Eurotiomycetes*	*Eurotiales*	*Trichocomaceae*	*Penicillium*	0.6
*Ascomycota*	*Eurotiomycetes*	*Sordariales*	*Cephalothecaceae*	*Cephalotheca*	0.6
*Ascomycota*	*Sordariomycetes*	*Orbiliales*	*Orbiliaceae*	*Arthrobotrys*	0.5
*Ascomycota*	*Orbiliomycetes*	*Peltigerales*	*Peltigeraceae*	*Peltigera*	0.4
*Ascomycota*	*Lecanoromycetes*	*Eurotiales*	*Trichocomaceae*	*Eurotium*	0.4
*Basidiomycota*	*Urediniomycetes*	*Sporidiobolales*	*Sporidiobolaceae*	*Rhodotorula*	0.1
*Ascomycota*	*Sordariomycetes*	*Xylariales*	*Xylariaceae*	*Thielavia*	0.1
*Ascomycota*	*Sordariomycetes*	*Microascales*	*Microascaceae*	*Verticillium*	0.09
*Basidiomycota*	*Sordariomycetes*	*Russulales*	*Stereaceae*	*Acanthophysium*	0.07
*Basidiomycota*	*Agaricomycetes*	*Polyporales*	*Polyporaceae*	*Trametes*	0.06
*Basidiomycota*	*Agaricomycetes*	*Agaricales*	*Schizophyllaceae*	*Schizophyllum*	0.06
*Basidiomycota*	*Agaricomycetes*	*Polyporales*	*Polyporaceae*	*Polyporus*	0.05
*Basidiomycota*	*Agaricomycetes*	*Agaricales*	*Schizophyllaceae*	*Chrondrostereum*	0.03

a Thirty predominant fungal genera shown in Figure 3.

b Percent of sequence reads in all libraries (27,987 total).

Each compost had a unique community composition and community dynamic. In the coffee compost, *Fusarium, Aspergillus*, and *Eurotium* were the predominant genera in the mesophilic phase library and accounted for 59, 18, and 6% of the sequences, respectively (Figure 3). In the thermophilic stage, *Fusarium* and *Aspergillus* remained the most abundant and accounted for 69 and 4%, of the sequences, respectively (Figure 3). However, *Eurotium* was displaced by *Monascus* (9%, Figure 3). In the mature stage, a new set of genera predominated, which included *Chaetomium, Monacrosporium, Scytalidium*, and *Hyaloscypha* (Figure 3). In the rice compost, three genera, *Scytalidium, Chaetomium*, and *Fusarium*, were predominant throughout. Of these, *Scytalidium* was consistently most abundant, and accounted for 52, 21, and 22% of the sequences in the mesophilic-, thermophilic-, and mature-stages, respectively (Figure 3). In the mesophillic bagasse compost, *Scedosporium* and *Fusarium* accounted for most of the sequences (42 and 38%, respectively). But, by the end of the process, these genera were replaced by *Scytalidium, Chaetomium*, and *Gleophyllum* (Figure 3). It should be noted that, as with any environmental analysis, spatial variability in the composts could affect the results such that the community composition of any given sample could differ to some extent from any other sample. Thus, the aforegoing discussion of the communities determined as representative of the compost types and phases is presented with this caveat.

### Potential pathogens in composts

Fifteen known fungal human pathogenic species were identified, and included seven that have been reported by prior investigators in composts (*Alternaria alternata, Aspergillus fumigatus, Candida tropicalis, Chaetomium funicola, Cladosporium cladosporioides, Fusarium oxysporum, Scytalidium lignicola*) as well as eight that have not been previously reported in such systems (*Bipolaris spicifera, Fonsecaea pedrosoi, Metarhizium anisopliae, Retroconis fusiformis, Scedosporium apiospermum, Scedosporium aurantiacum, Scedosporium prolificans, and Cladophialophora arxii*). Seven of these species (*Alternaria alternata, Aspergillus fumigatus, Chaetomium funicola, Cladosporium cladosporioides, Fusarium oxysporum, Scedosporium aurantiacum*, and *Cladophialophora arxii*) were identified in all composts, five species (*Metarhizium anisopliae, Retroconis fusiformis, Scedosporium apiospermum, Scytalidium lignicola*, and *Scedosporium prolificans*) were present in both bagasse and coffee composts, and three were detected in bagasse alone (*Bipolaris spicifera, Fonsecaea pedrosoi, and Candida tropicalis*).

## Discussion

The rank-abundance data showed that, in the coffee system, diversity decreased between the mesophilic and thermophilic phases, and then increased to its highest levels in mature phase. The rice compost showed a contrasting pattern with diversity increasing to its maximum level in the thermophilic phase, and then declining in the mature phase. The bagasse system, which lacked a thermophilic phase, showed the greatest expansion in fungal diversity from the mesophilic to mature stage, and thus achieved the highest level of diversity overall. These findings supported the concept of fungal community successions occurring across compost phases (Ryckeboer et al., 2003). But, the data did not consistently support prior findings that the occurrence or extent of a thermophilic phase to be the most important factor affecting the levels of diversity in the finished product (mature phase).

Previous studies have shown that fungal diversity decreased at thermophilic temperatures (Ryckeboer et al., 2003; Tiquia, 2005; Bonito et al., 2010; Hultman et al., 2010). But for the composts which attained thermophilic temperatures in the present study (rice and coffee), only the coffee was consistent with this finding. A possible explanation for the divergent behavior of fungal communities in these composts could have been the level of heat generated and duration of the thermophilic phase. In this regard, the coffee compost reached and maintained 64°C for 5 d, while the rice system heated to 57°C for 3–4 d. Notably the bagasse system, which never attained a thermophilic temperature exhibited the greatest expansion in fungal diversity. This could reflect the fact that most terrestrial fungi are mesophiles (Dix and Webster, 1995), and maintenance of mesophilic conditions in the bagasse could have allowed their undisrupted growth, and consequently development of communities more diverse than those of the other composts. Previously we reported analysis of prokaryotic diversity in the same bagasse, coffee, and rice composts (De Gannes et al., 2013), and comparison of that data with the present study shows that prokaryotic diversity was far greater than that of fungi. This finding was congruent with those of other investigators (Ryckeboer et al., 2003; Tiquia, 2005).

The prevalence of *Fusarium* might be anticipated given its ubiquity both in soil and association with plants. But, the relative abundance of *Fusarium* may to some extent have been an indicator of compost maturity, as its sequences tended to be greatest in meso- and thermo-philic phases (Table S2). Thus, mature phase compost was characterized by a reduction in *Fusarium*. All *Fusarium* sequences were matched to *Fusarium oxysporum* (Table S2), strains of which exhibit a variety of activities that range from lignin degradation (Kadarmoidheen et al., 2012) to causing plant (Beckman, 2000), animal (Odds et al., 1998), and human diseases (Boutati and Anaissie, 1997). *Scytalidium* was also prevalent in the composts, and the two species identified, *Scytalidium thermophilum* and *Scytalidium lignicola*, were divergently distributed (Table S2). The former of these was predominantly associated with the coffee and rice composts, and in the coffee system showed a striking increase in abundance in the mature phase (Table S2). Prior investigators have noted that this thermophilic fungus often exhibits profuse growth in mature phase compost from spores produced during thermophilic phase growth (Wiegant, 1992). In contrast, the majority of *Scytalidium lignicola* sequences were identified in the mature phase of the bagasse compost (Table S2), which lacked a thermophilic phase. The two species of *Chaetomium* identified, *Chaetomium funicola* and *Chaetomium globosum*, showed a similar bipartite distribution with the thermophilic organism, *Chaetomium funicola*, occurring primarily in rice and coffee composts (especially the coffee mature phase) while abundance of the mesophilic *Chaetomium globosum* was heavily weighted toward the mature phase bagasse (Table S2).

Prior studies have provided insights about the presence of opportunistic pathogens associated with composting systems, *viz*. *Candida tropicalis* and *Candida krusei* (Bonito et al., 2010), *Aspergillus fumigatus* (Dehghani et al., 2012), *Scytalidium lignicola*, and *Alternaria alternata* (Anastasi et al., 2005). However, the deep sequencing presented here has revealed the broadest spectrum of potentially pathogenic fungal species yet reported for a composting study. The group of eight species detected in all composts includes all but one of the potential pathogens identified by prior investigators, and thus could be representative of the main types of potential pathogens that might be encountered in composts. If so, bio-monitoring efforts could be expanded beyond *Aspergillus fumigatus* to include these organisms as well.

Fungal pathogens are an increasing threat to human health, and those listed above are known to cause several types of mycoses, with immune-compromised individuals being particularly susceptible. Allergic broncho-pulmonary aspergillosis, caused primarily by *Aspergillus fumigatus* (Latge, 1999; Chakrabarti et al., 2002; Hope et al., 2005; Agarwal et al., 2009) is a wide-spread concern (Howard et al., 2010; Denning et al., 2011). Skin diseases are also common and include phaeohyphomycosis caused by *Scytalidium lignicola, Candida tropicalis* (Wingard et al., 1979), *Scedosporium prolificans* (Revankar et al., 2002), and *Bipolaris spicifera* (McGinnis et al., 1992) as well as chromoblastomycosis, which have involved *Fonsecaea pedrosoi* and *Chaetomium funicola* (Pipenbring et al., 2007). The latter affliction is common in tropical and subtropical regions (Silva et al., 2002).

The clinical spectrum for most of these fungal pathogens is diverse. For example, *Bipolaris spicifera* is a source of nosocomial infections and has also caused a type of meningitis (Ogden et al., 1992) as well as cutaneous and pulmonary infections (McGinnis et al., 1992). *Scedosporium prolificans* have been shown to cause ocular infections (keratouveitis; Arthur et al., 2001) as well as cases of pneumonia (Berenguer et al., 1997); treatment of *Scedosporium prolificans* infections are difficult because of its resistance to many commonly used anti-fungal agents. *Scedosporium apiospermum* causes a range of infections such as bullous and necrotic purpura (Miyamoto et al., 1998), osteomyelitis (German et al., 2004) while infection by *Scedosporium aurantiacum* can result in malignant otitis externa, osteomyelitis, invasive sinusitis, keratitis, and pneumonia (Alastruey-Izquierdo et al., 2007). Concerns with *Fusarium oxysporium* include production of a range of mycotoxins (trichothecenes, zearalenon, and fumonisins) in addition to infections ranging from skin lesions to allergies (Boutati and Anaissie, 1997). *Alternaria alternata* also produces mycotoxins that caused allergies and asthma in children (Halonen et al., 1997). Infections caused by *Cladophialophora arxii* are chronic and appear as spreading mycoses of the skin, subcutaneous tissues, and nail (Barde and Singh, 1984).

According to the American Biological Safety Association, fourteen of the fungal pathogens identified in this study are categorized as Biosafety Level 2 (*Alternaria alternate, Aspergillus fumigatus, Candida tropicalis, Chaetomium funicola, Cladosporium cladosporioides, Fusarium oxysporum, Scytalidium lignicola, Bipolaris spicifera, Fonsecaea pedrosoi, Metarhizium anisopliae, Retroconis fusiformis, Scedosporium apiospermum, Scedosporium aurantiacum, Scedosporium prolificans*) and one (*Cladophialophora arxii*) belonged to Biosafety Level 3. Hence, it is recommended that personal protective equipment be worn when handling these composts. These include face protection (goggles, face masks), disposable gloves, and protective coats. It should be noted that, for all of the foregoing species, there can exist substantial intra-specific (strain level) variation in virulence (Ben-Ami et al., 2010). Thus, molecular detection such as that of the present study can provide an indicator of the potential health threat, but additional bioassays of isolates are needed to assess virulence.

## Conclusions

Results of the study supported both hypotheses. First, the community structure of each system was unique throughout the composting process, and did not converge on a common composition in the finished product. Thus, there was no single fungal community structure that could be considered typical. Second, the diversity of potential pathogens was greater than previously reported. Molecular identification of these indicated that safety precautions would be warranted for both compost producers and users. Overall, the study demonstrated that deep sequencing can effectively elucidate fungal community diversity in compost and that such information can have important implications for compost use and human health.

## Conflict of interest statement

The authors declare that the research was conducted in the absence of any commercial or financial relationships that could be construed as a potential conflict of interest.
